# Telerehabilitation for Post-Hospitalized COVID-19 Patients: A Proof-of-Concept Study During a Pandemic

**DOI:** 10.5195/ijt.2021.6383

**Published:** 2021-06-22

**Authors:** Pamela Tanguay, Nicole Marquis, Isabelle Gaboury, Dahlia Kairy, Matthieu Touchette, Michel Tousignant, Simon Décary

**Affiliations:** 1 École De Réadaptation, Université De Sherbrooke, QC, Canada; 2 École De Réadaptation, Université De Montréal, QC, Canada; 3 Faculté De Médecine Et Des Sciences De La Santé, Université De Sherbrooke, QC, Canada; 4 Centre De Recherche Sur Le Vieillissement, Ciusss-Iugs, QC, Canada; 5 Tier 1 Canada Research Chair In Shared Decision Making and Knowledge Translation, Vitam — Centre De Recherche En Santé Durable, QC, Canada

**Keywords:** COVID-19, Physical Therapy, Pulmonary, Rehabilitation, Telerehabilitation

## Abstract

**Purpose:**

Telerehabilitation could prevent sequelae from COVID-19. We aimed to assess the feasibility of telerehabilitation; describe pulmonary and functional profiles of COVID-19 patients; and explore the effect of telerehabilitation on improving pulmonary symptoms and quality of life.

**Methods:**

We conducted a pre-experimental, pre-post pilot study. We recruited COVID-19 patients who had returned home following hospitalization. The intervention included eight weeks of supervised physiotherapy sessions. We documented technological issues, success of recruitment strategies, and participants' attendance to supervised sessions. We measured the impact of pulmonary symptoms on quality of life and functional health.

**Results:**

We scheduled 64 supervised sessions with seven participants with few technological issues. Initial scores showed that pulmonary symptoms moderately to highly impacted quality of life. At eight weeks, all patients had improved from 10 to 45 points on the EuroQol-Visual Analog Scale (EQ-VAS) instrument, indicating clinical significance.

**Conclusion:**

We developed and administered a telerehabilitation intervention during a global pandemic that targets key symptoms of the relevant disease.

Since March 2020, the initial response from countries worldwide aimed to strengthen hospital-based care to prevent health systems collapse and death of infected patients of COVID-19. In June 2020, the World Health Organization declared that rehabilitation was the most disrupted healthcare service (World Health Organization, 2020). Yet, it has been declared by a consensus statement that rehabilitation is vital to recover from COVID-19 (Barker-Davies et al., 2020). The rehabilitation community has rightfully focused its current efforts on delivering pulmonary rehabilitation in acute, hospital or intensive care settings (Marta Lazzeri et al., 2020; Pedersini et al., 2020; Thomas et al., 2020). Long duration of hospital treatments can cause “post-intensive care syndrome” characterized by worsening physical disability and cognitive impairments due to immobilization, muscle wasting, and thoracic pain related to the use of a respirator (Thomas et al., 2020).

We have not yet grasped the long-term impact of COVID-19 on rehabilitation needs. Previous evidence in other strains of severe acute respiratory syndrome (SARS) viruses shows that patients exhibit impaired lung and physical function and reduced quality of life years after an infection (BMJ Best Practice, 2021; Moldofsky & Patcai, 2011; Zhang et al., 2020). COVID-19 has a major impact on lungs, potentially resulting in severe bilateral pneumonia requiring intensive care (Huang et al., 2020). Rapidly expanding evidence shows that COVID-19 likely impacts multiple body systems (Chow et al., 2020; Oxley et al., 2020). A recent analysis of 143 participants in Italy found that 44% of patients report worsened quality of life two months after returning home from hospitalization (at least ten points on EuroQol-Visual Analog Scale [EQ-VAS]) (Carfì et al., 2020). Eighty-seven percent of patients reported persistence of at least one symptom including fatigue, dyspnea, or joint pain (Carfì et al., 2020). These results indicate potential long-term sequelae of COVID-19.

The rehabilitation community is exploring new models of care to prevent long-term sequelae from COVID-19. The Stanford Consensus Statement recommends individualized rehabilitation that focuses on relieving symptoms of dyspnea and improving physical function and quality of life (Barker-Davies et al., 2020). In addition, rehabilitation of COVID-19 should focus on education and development of self-management skills (Barker-Davies et al., 2020). The Centers for Disease Control and Prevention (CDC) framework for primary care following a pandemic advocate that rehabilitation should be a priority for patients recovering from an infection (Krist et al., 2020). To comply with the public health recommendation on social distancing, delivering rehabilitation remotely appears to be the most feasible option.

Telerehabilitation is an ideal method of healthcare delivery in this context because it can provide safe, long-distance services; adhere to the sanitary measures required by the pandemic; and cover a large geographic region. Telerehabilitation also protects clinicians since patients may still be infectious for days following hospital discharge. Finally, pulmonary telerehabilitation was shown to be equivalent with in-person pulmonary rehabilitation (Marquis, Larivée, Dubois et al., 2014; Marquis, Larivée, Saey et al.., 2015; Tousignant et al., 2012; Vasilopoulou et al., 2017). In this paper, we identify gaps in providing telerehabilitation for COVID-19 patients after hospitalization.

We aimed to (1) assess the feasibility of providing telerehabilitation for COVID-19 patients after hospitalization; (2) describe pulmonary and functional profiles of COVID-19 patients; and (3) describe the effect of the intervention on improving pulmonary symptoms and quality of life.

## METHODS

### ELIGIBILITY AND RECRUITMENT

We aimed to design a safe virtual rehabilitation intervention that would help patients' recovery following hospitalization for COVID-19. We would follow up to ten patients for this proof-of-concept study. We included participants who received a diagnosis of COVID-19 (confirmed by hospital health professional) and were hospitalized due to COVID-19 or stayed in intensive care. Eligible participants had returned to their place of residence post hospitalization. Participants also had access to a device that had a camera and could connect to the Internet, were considered medically stable by an attending physician to participate in our intervention, and were able to follow instructions remotely.

As the study was ongoing, people in the community having COVID-19 started experiencing persistent symptoms which led to the term “Long-COVID.” Long-COVID is defined as the presence of signs and symptoms four weeks or longer after the infection of COVID-19 (National Institute for Health and Care Excellence, 2020). To help us understand community-based needs for patients who had COVID-19, one patient partner (participant seven) received our intervention. This participant signed a consent form and reported important symptoms and limited recovery but did not require hospitalization. Since her initial assessment did not differ from those of the other participants at the start of the intervention (see Table 3), we chose to include her results in our main analyses.

Participants were recruited by physicians and physiotherapists working in two COVID-19 designated hospitals. Study recruiters ensured that patients were hospitalized for symptoms related to COVID-19, and not for a comorbidity accentuated by COVID-19. This research was approved by the *Research Ethics Board of Centre intégré universitaire de santé et de services sociaux de l'Estrie – Centre hospitalier universitaire de Sherbrooke*. A research professional explained consent forms to patients via telephone interviews and obtained written consent via email from all participants. We completed the consent process without an in-person meeting, following public health safety recommendations.

### DEVELOPMENT OF THE COVID-19 VIRTUAL REHABILITATION CLINIC

Development of the COVIE-19 Virtual Rehabilitation Clinic began in April 2020. No data was available on the effectiveness of rehabilitation interventions in the community for patients with COVID-19. Based on discussion with intensive care physicians and physiotherapists and emerging international viewpoints and opinions (Barker-Davies et al., 2020), we hypothesized that the best evidence-based framework around which to design the intervention was pulmonary rehabilitation. Thus, we based our interventions on those of pulmonary rehabilitation programs (Spruit et al., 2013).

### ADAPTING EVIDENCE-BASED PULMONARY REHABILITATION PROGRAMS TO COVID-19

Although COVID-19 mainly affects the lungs (Barker-Davies et al., 2020), early observations showed that COVID-19 patients have other significant deficits such as fatigue and muscle pain (Carfì et al., 2020). These symptoms are also common in patients with lung diseases such as chronic obstructive pulmonary disease.

Pulmonary rehabilitation is a core non-pharmacological treatment for people with lung diseases (McCarthy et al., 2015) and is based on an exercise prescription for special populations (Garvey et al., 2016). Appendix A describes the key components of pulmonary rehabilitation and the components that we included in our program. We included breathing exercises, as these are often used during an exacerbation of pulmonary diseases to help correct alterations in respiratory patterns (Barker-Davies et al., 2020; Zadro et al., 2020). We included cardiovascular exercises in the form of mobilization-related interventions (e.g., walking) and goal-directed practice (e.g. stair training) (Zadro et al., 2020). We included strengthening exercises of the upper and lower body by targeted resistance training (e.g., sit-to-stand) or by using material available in a participant's home setting (e.g., weights, cans, bags of flour) (Zadro et al., 2020). We included multiple exercise components to address the multiple functional deficits of COVID-19.

Patient education on self-management skills is also a requirement of pulmonary rehabilitation to integrate behavioral changes that will foster a return to physical activities and improve physical function (Bourbeau & Nault, 2007). Indeed, a patient often completes most of the exercise dosage of any outpatient rehabilitation through unsupervised sessions at home. We addressed patient education during the warm-up and cooldown periods of the supervised sessions. These periods also allowed the physiotherapist to ensure the patient safely returned to his/her baseline physiological state.

We did not include content that addressed education on pulmonary medication, psychological counseling, or nutritional counseling from the pulmonary rehabilitation guidelines (Appendix A). Compared to diseases normally treated in pulmonary rehabilitation (e.g., COPD, pulmonary fibrosis), COVID-19 does not appear to be a chronic condition that requires long-term advice on nutrition or medication. However, future evidence may indicate a role for long-term advice. New evidence will also shed light on the psychological impact of having lived through COVID-19, and a new model may need to be developed to address interdisciplinary community-based rehabilitation.

To ensure the safety of each of the participants, the physiotherapist had the telephone number to reach the participant as well as his/her physical address to send first responders if necessary as well as the telephone number of the participant's caregiver. When available, the caregiver was present to further ensure safety and the exercises were carried out in a safe place (near a wall or a chair for support if necessary).

### DELIVERING THE TELEREHABILITATION INTERVENTION

Our telerehabilitation intervention is a physiotherapist-led intervention delivering a pulmonary telerehabilitation program in two stages for a suggested follow-up at eight weeks (Burge et al., 2020; Hansen et al., 2020; McCarthy et al., 2015; Vasilopoulou et al., 2017). The intervention was given by a physiotherapist who has experience with respiratory diseases and with the advice of a professor with 15 years of clinical experience in chronic respiratory diseases as well as research expertise in telerehabilitation for this population.

#### PHASE ONE: INTENSIVE REHABILITATION AND EDUCATION

We provided two weekly supervised sessions of breathing, cardiovascular, and strengthening exercises and intensive patient education on self-management skills (Bourbeau & Nault, 2007). Since COVID-19 may produce heterogeneous symptoms, our intervention was individualized according to the participant's needs (Barker-Davies et al., 2020). We matched the dosage of the exercise to patient's main goals and symptoms. For example, some participants needed more cardiovascular exercises because their dyspnea more significantly impacted their quality of life, while others might have required more strengthening exercises to counteract muscle weakness. The physiotherapist personalized each exercise to ensure optimal dosage and loading. Participants were advised to complete an additional 30-minute unsupervised cardiorespiratory and breathing exercise session daily and a one-hour unsupervised strengthening exercise session per week.

#### PHASE TWO: CONSOLIDATION

We provided once-weekly supervised sessions for consolidation of self-management skills. Participants were advised to complete one-each additional 30-minute unsupervised cardiovascular and breathing exercise sessions daily and two one-hour unsupervised strengthening exercise sessions per week. By gradually decreasing the number of supervised sessions per week, we intended to develop the self-management skills necessary for patients to return safely to their usual activities. We continued phase one as long as the participant's condition required a follow-up of two sessions per week until the participant was ready to move to phase two. Each participant could receive up to 16 one-hour supervised sessions over eight weeks.

### TECHNOLOGY

The infrastructure includes a clinical management system focused on telerehabilitation with videoconferencing components (i.e., camera, speakers, microphone). The software environment consists of two interfaces that combine user-friendly and optimal control of the technology to enhance the telerehabilitation experience for both the participant and the clinician. A web link is sent to the patient to activate the session. The software is a cloud-based multi-point, multi-view, and multi-stream (video and audio) telecommunication system. This technology is freely available upon request to the authors of this study. The platform used for the telerehabilitation sessions was built by the research team in previous work and the code is Open Source.

### FEASIBILITY OUTCOMES

We documented four aspects of feasibility. First, although our team has developed and used the Tera+ platform in numerous telerehabilitation trials, tracking technological and server issues remains essential to offer telerehabilitation broadly. The physiotherapist delivering the intervention kept a logbook of all technological issues that happened during consultations throughout the project and worked with an engineer to resolve them in a timely matter.

Second, one of the most complex issues facing community-based clinical research teams worldwide is the capacity to recruit participants during a global pandemic. During recruitment, the research team tracked the referrals of each professional to determine the most effective recruitment techniques and settings. We also considered the recruitment flow (i.e., number of potential participants received per week) and time required to reach the desired sample size of ten participants.

Third, we documented adverse events during the intervention (falls or injuries) and we documented (self-reported by participants) what other symptoms the participants had (vertigo, loss of balance, loss of smell, dizziness).

Finally, we measured the attendance rate by the number of sessions attended as per the originally scheduled date, and we tracked the number of absences. We documented the number of weeks for each of the two phases (intensive rehabilitation and education and consolidation). We measured satisfaction with our intervention with a questionnaire developed from the adaptation and translation of the Patient Satisfaction Questionnaire for Telemedicine (Yip et al., 2003). This questionnaire consists of 15 statements rated on a five-level Likert scale where one represents “strongly disagree” and five represent, “strongly agree.” Scores of 70 out of 75 represent high satisfaction.

### CLINICAL OUTCOMES TO CHARACTERIZE PULMONARY AND FUNCTIONAL PROFILES

#### CORE OUTCOME SET

No core outcome set exists to study the rehabilitation of COVID-19 patients. We needed easy-to-use measures that addressed domains relevant to assessing pulmonary symptoms and functional impacts on quality of life.

Table 1 describes our three standardized tools to assess pulmonary symptoms, quality of life and physical activity levels. We administered the EuroQol-5D-5L (EQ-5D-5L) and EQ-Visual Analog Scale (VAS) to assess functional health and quality of life (EQ-5D, 2020). The tool consists of five dimensions: mobility, self-care, usual activities, pain/discomfort and anxiety/depression. Each question is scored on a one-to-five-point scale, where one represents no impact on quality of life. The EQ-VAS is a scale from 0 to 100 where 0 represents the “worst imaginable health” and 100 the “best imaginable health”. In the context of COVID-19, the minimum clinically important difference (MCID) for the EQ-VAS is proposed as ten points (Carfì et al., 2020).

**Table 1:** Core Outcome Set to Assess Pulmonary and Quality of Life Domains of COVID-19 Patients

Measure	Number of items	Response format	Range of score	Score interpretation	MCID
COPD Assessment Test (Polkey et al., 2018)	8	5-point Likert scale	0–40	5: No impact 10: Low impact 10–20: Medium impact 20–30: High impact 30: Very high impact	2 or more points (Kon et al., 2014)
EQ-5D-5L (*EQ-5D*, 2020)	5	5-point Likert scale from “no problems” to “unable to/extreme problems”.	0–5	Higher score = worst health	Not reported
EQ-VAS (*EQ-5D*, 2020)	1	100-point visual analog scale from “The best health you can imagine” to “the worst health you can imagine”	0–100	Higher score = better health	EQ-VAS : 10 points(Carfì et al., 2020)

We administered the COPD Assessment Test (CAT) to assess the severity of pulmonary symptoms and their impact on quality of life (Polkey et al., 2018). The CAT consists of eight questions and each question is scored from zero to five points for a maximum score of 40 points. Higher scores represent a decrease in quality of life due to pulmonary symptoms. A difference of two points is considered a clinically significant change (Kon et al., 2014).

#### ADDITIONAL CLINICAL OUTCOMES

We assessed four additional outcomes to describe pulmonary symptoms. We assessed physical activity levels using the Baecke Physical Activity Questionnaire (Pitta et al., 2006; Troosters et al., 2010). The questionnaire assesses three domains: occupational activities, sports activities, and recreational activities. Questions related to sports activities were adapted to the activities allowed during the lockdown (e.g., walking, yoga, home training, running). General physical activity is the summation of the score of the three domains. The score ranges from three to 15 points, with higher scores indicating higher physical activity participation.

We evaluated cough categorized as strong or weak, productive or not, dry or wet, effective or not. A normal cough is considered to be strong, not productive, dry and effective. We assessed the sensation of dyspnea with the modified Borg scale (0–10). A high score indicates a higher sensation of shortness of breath whereby an improvement of one point is considered clinically significant (Ries, 2005). Finally, we assessed pulmonary secretions including frequency, intensity, quantity (number of times/day), appearance (thick or liquid) and color. We measured body pain using a 10-point visual analog scale. Higher scores indicate higher levels of body pain and a difference of two points is considered a MCID (Ries, 2005).

#### DATA COLLECTION AND ANALYSIS

An independent research coordinator who did not deliver the intervention collected outcomes. At the end of each evaluation, the independent researcher gave the results to the physiotherapist in charge of the intervention to individualize the sessions according to the remaining deficiencies. The independent researcher collected all outcome measures online via the TeRA+ telerehabilitation platform. Data were collected twice: at the beginning of the intervention, and eight weeks later. We used frequency counts and descriptive statistics to analyze feasibility outcomes and pulmonary and functional profiles at baseline. To explore the impact of the intervention, we assessed whether each patient reached the MCID in outcomes between the beginning and end of the sessions.

## RESULTS

### PARTICIPANTS

Of the ten participants we attempted to recruit, we were able to recruit seven participants. Only one participant did not complete the intervention since he required re-hospitalization. Characteristics of participants are described in Table 2. Participants were between 49 and 80 years of age and three of seven were women. Participants reported having high blood pressure (3/7 participants), hypercholesterolemia (3/7) and asthma (2/7). Participants' body mass indices ranged from 21 kg/m^2^ to 39 kg/m^2^.

**Table 2:** Characteristics of Participants

Participants	Age	Sex	Comorbidity	BMI (kg/m^2^)	Access to caregiver	Phase 1 (number of weeks)	Phase 2 (number of weeks)	Number of supervised sessions	Intensive care stay
1	49	M	HBP, HC	21.5	Yes	3	2	8	Yes
2	56	F	Asthma, HC, HT_4_	32.4	No	1	2	4	No
3	80	M	DB, Asthma	28.6	Yes	1	N/A	2	Yes
4	59	M	N/A	30.9	No	5	3	13	Yes
5	64	M	HBP, HC	23.9	No	7	1	15	Yes
6	53	F	HBP, KD	39.3	Yes	8	0	16	Yes
7	49	F	N/A	27.0	No	1	4	6	No

*Note.* M: Male; F: Female; HBP: high blood pressure; HC: hypercholesterolemia; HT_4_: Hypothyroidism; DB: Diabetes; KD: Kidney disease

### FEASIBILITY OUTCOMES

#### TECHNOLOGICAL ISSUES

We identified four categories of technological issues during the intervention. (1) In 34% of sessions, we experienced lack of synchronization between audio and video caused by a slow Internet connection. (2) In 19% of sessions, notifications from other applications on mobile devices interrupted the session and required participants to reconnect to continue the session. (3) In three percent of sessions, participants could not connect because they either used an unsupported Internet browser for Tera+ (Tera+ is supported only by Google Chrome or Safari), they lost the Internet connection, or they experienced difficulty connecting to the platform. (4) Participants had issues activating their microphone and camera each time they connected to the platform. Some participants could solve issues by themselves while others required help from the physiotherapist. All the sessions were delivered even with the four issues mentioned.

#### RECRUITMENT PROCESS

We centered our recruitment strategy on identifying all patients referred to our two COVID-19 designated hospitals within our administrative region. We identified one physician for each site and the physiotherapist's team of *Centre hospitalier universitaire de Sherbrooke*. We found that they independently triaged patients to be recruited to our project. The physiotherapist team referred eleven participants and physicians did not send any candidates.

For the recruitment flow, we received on average two or three candidates each week in the first month (May), and one additional participant in the first week of June. It took two months (early May to late June) to recruit seven participants. The number of new cases admitted to COVID-19-designated hospitals rapidly fell as our region implemented stringent public health safety measures. To increase our recruitment capacity, we aimed to offer our intervention to other administrative regions since the intervention was delivered remotely. However, we faced communication issues with these new COVID-19 hospitals, and limitations from the ethics approval process. For these reasons, we decided to close recruitment after two months.

Two contacted participants refused to take part in the project and three did not have the necessary technology (i.e., computer with a camera, tablet, or smartphone). Our recruitment rate was 55% (6/11). The research team identified a patient partner (participant seven) to assess community-based rehabilitation needs, and we included this patient in our recruitment sample.

#### ATTENDANCE AT SUPERVISED SESSIONS

All participants attended the supervised sessions scheduled by the physiotherapist. We delivered 64 supervised sessions. The total number of supervised sessions per participant ranged from four to 16 with a median of eight. Participants postponed only two out of 64 sessions (attendance rate: 97%). The duration of the two phases for each participant and the number of supervised sessions are described in Table 2.

#### SAFETY AND SATISFACTION

No serious adverse events were reported during or after the sessions in this limited sample. Satisfaction scores ranged between 71 and 74 out of 75, representing high satisfaction for all participants.

### CLINICAL OUTCOMES

#### PULMONARY AND FUNCTIONAL PROFILES

Table 3 describes the pulmonary and functional profiles of the participants before the intervention. Initial CAT scores showed that pulmonary symptoms moderately impacted quality of life in five participants (scores between 10 to 20/40). One participant was highly impacted (21/24) and one experienced a very high impact on quality of life (38/40). The EQ-VAS ranged between 25 and 70 points with a median of 50 points. The dimensions of EQ-5D-5L were heterogeneously impacted. The range of scores for self-care was one to five. For usual activities, this metric ranged from three to five; for mobility it ranged from one to four; for pain/discomforts it ranged from one to four; and for anxiety/depression from one to three. Five participants had a low level of physical activity (score <7.5) before the intervention, and two had a satisfactory level (score ≥9). Appendix B describes levels of physical activity for the three dimensions (occupational activities, sports activities and recreational activities).

**Table 3:** Pulmonary and Functional Profiles before the Intervention

Participants	CAT	EQ-VAS	EQ-5D-5L					Baecke Physical Activity Questionnaire	Pain		Pulmonary symptoms		
			Mobility	Self-care	Usual activities	Pain/discomfort	Anxiety/depression		At rest	On Exertion	Cough	Secretion	BORG
1	11	70	2	1	3	1	1	6.0	0	0	NTR^*^	None	7
2	38	30	3	3	3	2	3	9.1	5	8	Wet^*^	Thick, green	8
3	21	60	3	5	5	1	3	4.9	0	0	NTR^*^	Thick, green	8
4	14	60	4	4	5	4	3	7.0	0	0	NTR^*^	None	7
5	10	50	3	2	3	4	1	6.9	0	2	NTR	Liquid, white	8
6	16	25	3	2	4	4	2	6.4	0	4	Weak, non-effective^*^	Can't expectorate	6
7	18	40	1	2	3	2	1	9.8	4	8	Wet^*^	None	7
min. – max.	10–38	25–70	1–4	1–5	3–5	1–4	1–3	6.0–9.8	0–5	0–8	N/A	N/A	6–8

*Note.* CAT: COPD Assessment Test; For the physical activity: <7.5 represented Low levels of physical activity/[7.5–9.0 [: Moderate level/≥9: Satisfactory level; NTR: Nothing to report, the cough is strong, effective and dry;

* exercise induced cough

Four participants had pain located in the neck or shoulder because of COVID-19. One participant had pain in the leg caused by a nerve compression that occurred during an intensive care stay. Two participants had a wet cough; one participant had a weak cough; and five participants had a strong, dry and effective cough. Six participants had a cough triggered by exercise. Two participants had thick and green secretions suggesting an infection. One participant was unable to expectorate, and one participant had liquid and white secretions. Three participants didn't have any secretion. One participant had dyspnea they ranked between six and eight out of ten (Borg scale) indicating difficulty in breathing during exercise.

Table 4 describes the pre-post changes for our main outcomes for each participant. For the EQ-VAS scores, the range of improvement was between 10 and 45 points, reaching the MCID (10 points). For the CAT score, five out of six participants improved from six to 13 points out of 40, reaching MCID (two points). At the end of the intervention, two participants had no impact of pulmonary symptoms on their quality of life (CAT score <5/40), two participants had a low impact (CAT score between 5 and 10/40), one participant had a moderate impact (CAT score 17/40) and one participant still had a high impact (CAT score 25/40). At the end of the intervention, two participants increased their score on the Baecke Physical Activity Questionnaire passing from a low level of physical activity to a moderate level. Two participants increased their scores by 0.2 and 1.0 points but were still considered at a low level of physical activity. The two participants maintained their satisfactory level through the end of interventions. Appendix B includes the preliminary impact of the intervention for the additional outcomes.

**Table 4:** Preliminary Impact of the Intervention on Core Outcomes Measures

	CAT			EQ-VAS		
Participant	Baseline	8 weeks	Delta	Baseline	8 weeks	Delta
1	11	3	8^*^	70	80	10*
2	38	25	13*	30	65	35*
3	21	–	–	60	–	–
4	14	8	6*	60	80	20*
5	10	4	6*	50	95	45*
6	16	6	10*	25	40	15*
7	18	17	1	40	70	30*
min.–max.	10–38	3–25	1–13	25–70	40–95	10–45

*Note.* CAT: COPD Assessment Test; Delta: absolute; ^*^: Achieve the MCID

Color code for CAT scores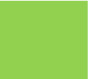 No impact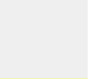 Low impact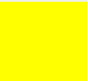 Moderate impact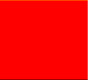 High impact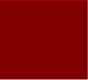 Very high impact

## DISCUSSION

We developed and safely delivered a fully online telerehabilitation intervention for patients returning home after a hospital stay for COVID-19. We adapted an evidence-based pulmonary telerehabilitation intervention to design this telerehabilitation intervention (Barker-Davies et al., 2020; Burge et al., 2020; Hansen et al., 2020; Vasilopoulou et al., 2017). This study helped us identify three important research gaps for future research.

### RECRUITMENT PROCEDURE

Our recruitment strategy was centered on recruiting patients from COVID-19-designated hospitals and was designed to meet the need for community-based rehabilitation. We worked in close collaboration with physicians and physiotherapists in COVID-19-designated hospitals. They identified the participants who were medically stable, able to follow instructions remotely, and safely participate in rehabilitation remotely. While ensuring safety, this strategy significantly limited recruitment. New cases rapidly declined in our administrative region and delays in ethics clearances occurred during summer to recruit patients from other hospitals and regions.

Moreover, limiting recruitment to hospitals was not optimal given current knowledge of COVID-19 patient profiles. Current evidence proposes four COVID-19 severity patterns: (1) Asymptomatic infected patients; (2) Symptomatic patients isolating at home; (3) Symptomatic patients admitted to a hospital and (4) Symptomatic patients requiring ventilatory support in critical care (Barker-Davies et al., 2020). Our strategy for the intervention only met the needs of patients in the last two groups. As new rehabilitation paradigms are emerging for the first two groups, they may also require pulmonary rehabilitation using a similar model to ours (Carfì et al., 2020; Greenhalgh et al., 2020).

Future studies should assess telerehabilitation for all symptomatic adults with persistent symptoms and delayed recovery (i.e., three weeks after infection) (Greenhalgh et al., 2020). There should be multiple recruitment channels focused on answering community-based needs. Telerehabilitation is adapted to the context of the pandemic and can deliver care remotely to almost any geographical region.

### FEASIBILITY RESULTS

Although technological issues with the Tera+ platform did not prevent sessions from happening, our records of technological issues indicate that healthcare professionals may need training before using the platform, as well as access to an efficient online support service. Technological issues did not appear to impact participants' satisfaction. All participants attended the scheduled supervised sessions, suggesting that participants perceived the intervention as very useful to their health. Participants received a median of eight supervised sessions by a physiotherapist over eight weeks. At the end of the intervention, participants were asked whether they would need further rehabilitation services after the eight weeks of our intervention. Five participants stated that eight weeks of rehabilitation was adequate; one would have preferred a longer follow-up. In future studies, patients requiring additional rehabilitation beyond eight weeks should be offered to continue rehabilitation outside of the research project.

### PULMONARY AND FUNCTIONAL PROFILES OF COVID-19 PATIENTS

We found that COVID-19 patients still faced moderate to severe pulmonary and functional disabilities when returning home after hospitalization. Clinical profiles of the participants were heterogeneous since some participants required several weeks in intensive care while others required only a short hospital stay. These differences may explain the wide range of disabilities across domains among our participants at the beginning of the intervention, but other factors may also explain these differences (e.g., pre-existing comorbidities, age, etc.). Participants who required an intensive care stay might have exhibited greater impairment of pulmonary symptoms (dyspnea on exertion). Within this intervention, some participants will remain in phase one longer, while others may move to the consolidation phase and the end of rehabilitation faster.

Our preliminary clinical results suggest that telerehabilitation may help participants recover from COVID-19. All of our participants increased their quality of life scores by at least 10 points. In contrast, recent evidence suggests that close to half of COVID-19 patients will experience a decrease of ten points in their quality of life after eight weeks without interventions (Carfì et al., 2020).

## LIMITATIONS

Our intervention is adapted from evidence-based pulmonary telerehabilitation, but we excluded components that may be required to achieve interdisciplinary community-based care as we learn more about long-term rehabilitation needs of COVID-19 patients. Our recruitment strategy was limited by the unique COVID-19 context because clinical teams within designated hospitals faced staff shortages and rapid turnover. Also, clinical settings faced daily to weekly changes in procedures due to frequently changing recommendations from government officials. Despite this context, we could build communication channels with attending physicians and physiotherapists and understand the environments of COVID-19 units. The results of this study are not generalizable to all patients recovering from COVID-19. Several COVID-19 rehabilitation subgroups likely exist. The lack of a control group and the small sample size precludes a precise assessment of the effectiveness of the intervention versus natural recovery. Another limitation of this study is that COVID-19 is a novel virus and there was no validated tool for this population when this study took place.

There was no serious adverse event in this study, but our sample size was small and new emerging evidence requires intensive effort to better recognize a wide array of post-COVID symptoms that patients may experience in the months following their hospitalization. Future research should emphasize a rigorous monitoring system for the dose, adherence, and fidelity of each exercise component to better describe exercise tolerance and intolerance in this population during the short-term rehabilitation period following a hospitalization.

## CONCLUSION

We developed and safely delivered a fully online telerehabilitation intervention for patients returning home after being hospitalized for COVID-19. We observed that patients recovering from COVID-19 showed heterogeneous pulmonary and functional profiles, and our participants experienced moderate to severe symptoms. Preliminary results showed that eight weeks of supervised physiotherapy sessions may improve symptoms, quality of life, and return to physical activities in COVID-19 patients.

### KEY MESSAGES

What is already known on this topic:

1. Evidence shows that COVID-19 likely impacts multiple body systems and can expose potential long-term sequelae.
2. Initial response from countries worldwide aimed to strengthen hospital-based care to prevent health systems' collapse and death of infected patients.
3. The ideal solution for COVID-19 would be to deliver rehabilitation remotely using technology to provide safe, physically distanced care.

What this study adds

1. We developed and safely delivered a fully online telerehabilitation intervention for patients returning home after being hospitalized for COVID-19.
2. We centered our recruitment procedure on COVID-19-designated hospitals and likely missed many patients in the community who may require rehabilitation to recover from COVID-19 although they did not require hospitalization.
3. We found that some patients recovering from COVID-19 exhibited moderate to high levels of pulmonary and functional disability.
4. Preliminary results showed that eight weeks of supervised physiotherapy sessions may improve pulmonary symptoms and quality of life in COVID-19 patients.

## APPENDIX A

**Table:** Components of the Telerehabilitation Intervention (Burge et al., 2020; Garvey et al., 2016; Hansen et al., 2020; McCarthy et al., 2015).

KEY COMPONENT OF PULMONARY REHABILITATION	DOSAGE	Intervention
Breathing techniques	Specific techniques to better control breathing and avoid feeling out of breath	✓
	Techniques to clear mucus	✓
Exercise training	Cardio Intensity should be between 50 and 70% of the heart rate reserve (Jones et al., 2015) 150 minutes of cardiorespiratory activity per week	✓
	Strengthen 3 times/Week	✓
Education	Education about the disease	✓
	Self-management	✓
	Medication	✗
Psychological counseling	Stress management to prevent depression or decrease anxiety	✗
Nutritional counseling	Recommendation about nutrition	✗

## APPENDIX B

**Table:** Supplemental Data

	EQ-5D-5L									
	MOBILITY		SELF-CARE		USUAL ACTIVITIES		PAIN/DISCOMFORT		ANXIETY/DEPRESSION	
Participant	Baseline	8 weeks	Baseline	8 weeks	Baseline	8 weeks	Baseline	8 weeks	Baseline	8 weeks
1	2	1	1	1	3	2	1	1	1	1
2	3	1	3	1	3	2	3	3	3	1
3	3	–	5	–	5	–	1	–	3	–
4	4	3	4	1	5	3	4	1	3	2
5	3	2	2	1	3	2	4	2	1	1
6	3	3	2	1	4	3	4	3	2	2
7	1	1	2	1	3	3	2	3	1	2
min. – max.	1–4	1–3	1–5	1–1	3–5	2–3	1–4	1–3	1–3	1–2

**Table:**

	PAIN					
	AT REST			ON EXERTION		
Participant	Baseline	8 weeks	Delta	Baseline	8 weeks	Delta
1	0	0	0	0	0	0
2	5	2	3	8	4	4
3	0	–	–	0	–	–
4	0	0	0	0	0	0
5	0	0	0	2	0	2
6	0	0	0	4	0	4
7	4	2	2	8	1	7
min. – max.	0–5	0–2	0–2	0–8	0–4	2–7

*Note.* Delta: absolute

**Table:**

	BAECKE PHYSICAL ACTIVITY QUESTIONNAIRE								
	OCCUPATIONAL ACTIVITIES			SPORTS ACTIVITIES			RECREATIONAL ACTIVITIES		
Participant	Baseline	8 weeks	Level of physical activity at T2	Baseline	8 weeks	Level of physical activity at T2	Baseline	8 weeks	Level of physical activity at T2
1	2.3	2.5	Moderate	1.5	1.8	Low	2.3	2.8	Satisfactory
2	3.1	3.8	High	2.3	2.3	Low	3.8	3.8	Satisfactory
3	2.1	–	–	1.3	–	–	1.5	–	–
4	2.8	2.9	Moderate	2.5	2.8	Satisfactory	1.8	2.5	Satisfactory
5	2.4	2.5	Moderate	2.3	2.0	Low	2.3	3.0	Satisfactory
6	2.1	2.4	Low	2.0	2.0	Low	2.3	2.3	Low
7	3.0	3.5	High	3.0	2.5	Satisfactory	3.8	3.8	Satisfactory
min. – max.	2.1–3.8	2.4–3.8	N/A	1.3–3.0	1.8–2.8	N/A	1.5–3.8	2.3–3.8	N/A

*Note.* Occupational activities: <2.5: low level; [2.5–3.1]: moderate level; >3.1: high-level; Sport and recreational activities: <2.5: low level; ≥2.5: satisfactory level

**Table:**

	PULMONARY SYMPTOM						
	COUGH		SECRETIONS		MODIFIED BORG SCALE		
Participant	Baseline	8 weeks	Baseline	8 weeks	Baseline	8 weeks	Delta
1	NTR^*^	NTR	None	None	7	2	5
2	Wet^*^	Greasy	Thick, green	Thick, yellow	8	7	1
3	NTR^*^	–	Thick, green	–	8	–	–
4	NTR^*^	NTR	None	None	7	4	3
5	NTR	NTR	Liquid, white	None	8	4	4
6	Weak, non-effective^*^	NTR	Can't expectore	None	6	4	2
7	Greasy^*^	NTR^*^	None	None	7	4	3
min. – max.	N/A	N/A	N/A	N/A	6–8	2–7	1–5

*Note.* NTR: Nothing to report, the cough is strong, effective and dry; ^*^exercise induced cough
